# Delayed Diagnosis of Wilson's Disease Report From 179 Newly Diagnosed Cases in China

**DOI:** 10.3389/fneur.2022.884840

**Published:** 2022-07-05

**Authors:** Minling Yu, Linxiang Ren, Muxin Zheng, Mingfan Hong, Zhisheng Wei

**Affiliations:** Department of Neurology, School of Clinical Medicine, The First Affiliated Hospital of Guangdong Pharmaceutical University, Guangzhou, China

**Keywords:** hepatolenticular degeneration, initial symptom, misdiagnosis, Wilson's Disease, delayed diagnosis

## Abstract

**Objective:**

To analyze the initial symptom and the cause of the misdiagnosis of Wilson's Disease (WD) so as to enhance awareness of this condition and reduce diagnostic errors.

**Methods:**

The clinical data of 179 patients with the confirmed diagnosis of WD who were hospitalized in the First Affiliated Hospital of Guangdong Pharmaceutical University from October 2014 to September 2021 were analyzed. Those patients who had attended two or more hospitals, had been misdiagnosed as other diseases, or failed to get a clear diagnosis for 3 months and over before hospitalization were included in the group of clinical misdiagnosis or the group without a definite diagnosis.

**Results:**

One hundred twenty-nine cases (72.1%) were misdiagnosed, 39 cases (21.8%) failed to be diagnosed as a specific disease, and only 11 cases (6.2%) had been diagnosed as WD within 3 months at the early stage of the disease. WD was easily masqueraded as a variety of diseases, including all types of hepatitis, cirrhosis, splenomegaly, hepatomegaly, encephalitis, encephalopathy, peripheral neuropathy, psychosis, osteoarthrosis, nephrosis, anemia, and other illnesses.

**Conclusion:**

Wilson's Disease is prone to long-term misdiagnosis or unclear diagnosis. Early diagnosis and treatment are the most important determinations of the prognosis. Therefore, when facing patients with doubtful WD, it is valued to perform Kayser–Fleischer ring, copper metabolism, imaging examination, genetic tests, and radioactive copper test if necessary.

## Introduction

Wilson's Disease (WD), also known as hepatolenticular degeneration (HLD), is an autosomal recessive genetic disease, mainly characterized as abnormal copper metabolism. It should be noted that the disease is rare, but patients may have access to achieve normal life expectancy through medical treatment if diagnosed and therapied timely (1). But patients usually tend to be misdiagnosed or missed diagnosis in the early onset, because of the wide spectrum of WD symptoms involve in different organs damage and lack specificity, easily making them lose the optimal timing of treatment (2). Therefore, it is imperative for us to summarize and analyze the reasons for misdiagnosis in clinical work in order to deepen awareness of WD and reduce diagnostic errors. In this study, we reviewed the clinical records of 179 patients with WD hospitalized in the Department of Neurology of the First Affiliated Hospital of Guangdong Pharmaceutical University from October 2014 to September 2021 and explore the causes of clinical misdiagnosis.

## Subjects and Methods

### Subjects

From October 2014 to September 2021, 179 with WD were hospitalized in the Department of Neurology, The First Affiliated Hospital of Guangdong Pharmaceutical University included 90 males and 89 female patients, aged 2–62 years, with an average of 24 (19, 32) years. All of them, from all over the country, were confirmed by wide spectrum of WD symptoms, copper biochemical, imaging examinations, as well as the genetic testing with sequencing of the *ATP7B* gene, meeting the diagnostic criteria for WD in Handbook Of Clinical Neurology 2017 (2).

### Method

#### Clinical Classification

The clinical classification of WD was performed by Yang Renmin's classified method (3), including cerebral type, visceral type, cerebral-visceral type, and bone-muscle type.

#### Group

Patients who had attended two or more hospitals and had been misdiagnosed as other diseases for more than 3 months after the first symptom appeared were included in clinical misdiagnosis group. Others failing to get a clear diagnosis for 3 months and over before hospitalization were classified as the undiagnosed group and those who could be diagnosed within 3 months were classified as timely diagnosed group.

The 179 patients with WD were divided into three groups: clinical misdiagnosis group, undiagnosed group, and the timely diagnosed group, which accounted for 129 (72.1%), 39 (21.8%), and 11 (6.2%), respectively. The first two groups accounted for 93.9% of the total.

#### Statistical Analysis

The statistical analysis was performed using SPSS 27.0 statistical soft–ware. The Kruskal—Wallis Test and chi-squared test were used to test the significance of the difference. Probability (*P*) values < 0.05 were considered to be statistically significant.

## Results

### Details in Clinical Misdiagnosis Group

Diagnostic errors were detected in 129 (72.1%) of the 179 patients (male: female = 67:62). The median (p25, p75) age at onset of symptoms in these patients was 26 (20, 33) years (range: 4–56 years old), with a 1 (0.6, 3)-year delay in diagnosis (range: 0.25–30 years). Of these, the initial manifestation of the 72 patients was characterized as non-cerebral symptoms (Table 1). The clinical classifications are as follows, cerebral type (*n* = 53), visceral type (*n* = 68), cerebral-visceral type (*n* = 4), and bone-muscle type (*n* = 4). A total of 53 cases (41.1%) were caused by cerebral symptoms, including 47 cases of extrapyramidal damage signs, such as dysarthria, salivation, limb shaking, muscle rigidity, torsion dystonia, chorea and athetosis, parkinsonism, atypical involuntary movements, etc., and six cases of mental and psychological symptoms, like mental retardation, cognitive decline, schizophrenia, manic depression, neurasthenia, somnipathy, etc. A total of 49 cases (38.0%) were caused by liver symptoms, including anorexia, jaundice, hepatosplenomegaly, ascites, abnormal liver function, etc., and 11 cases (8.5%) were caused by nephrotic symptoms, including edema, hematuria, proteinuria, etc. A total of 4 cases (3.1%) were caused by bone and joint symptoms, including bone and joint pain and limb movement disorders. Other symptoms occurred in 12 cases (9.3%), including abnormal blood cells, bleeding, hemolysis, peripheral neuropathy, etc. (Table 2). WD was easily masqueraded as multiple diseases, each patient was around misdiagnosed one or two times, a total of 139 times, and the name involved in organ systems including digestive (67/139), nerve (51/139), motion (4/139), urinary (5/139), mental (5/139), blood (3/139), endocrine metabolism (2/139), gynecology (2/139), among which, the most common specific diseases misdiagnosed were hepatitis (32/139), liver cirrhosis (21/139), extrapyramidal diseases (47/139), various kinds of nephritis, nephropathy (5/139), arthritis (4/139), mental and psychological diseases (5/139), and other diseases (25/139).

**Table 1:** Comparing age of onset, time of diagnosis, and initial symptoms among three groups.

**Group**	**Case**	**Age of onset** **(years)**	**Time from onset to diagnosis** **(years)**	**Non-cerebral initial symptoms (case) (proportion)**
Clinical misdiagnosis group	129 (72.1%)	26 (20, 33)	1 (0.6, 3)	72 (55.8%)
Undiagnosed group	39 (21.8%)	21 (16, 29)	1 (0.6, 2)	11 (28.2%)**
Timely diagnosed group	11 (6.2%)	14 (5, 38)	0.08 (0.04, 0.17) **	9 (81.8%)
H(K)/χ^2^		1.552	23.004	13.483
*P*-Value		0.460	<0.001	0.001

vs. clinical misdiagnosis group, ** P < 0.01; Age of onset and Time from onset to diagnosis use Kruskal–Wallis Test; non-cerebral initial symptoms use chi-squared test.

### Details in Undiagnosed Group

Among the 39 cases, 17 were male and 22 were female. The age ranged from 4 to 53 years, with an average of 21 (16, 29) years. The time from onset to diagnosis was 1 (0.6, 2) year, and 11 cases had the first non-cerebral symptoms (Table 1). Clinical classification: cerebral type 28 cases, visceral type 11 cases. Initial symptoms: cerebral symptoms in 28 cases (71.8%), all of which were symptoms of extrapyramidal system damage; nine cases (23.1%, mainly liver dysfunction, jaundice and poor appetite) were diagnosed with liver symptoms and 2 cases (5.1%) with kidney symptoms (Table 2).

**Table 2:** Comparing clinical classification, initial symptoms among three groups.

**Clinical classification and initial symptom**		**Clinical misdiagnosis group (*n* = 129)**	**Undiagnosed group** **(*n* = 39)**	**Timely diagnosed group** **(*n* = 11)**	**χ^2^**	***P*-Value**
Clinical classification	Cerebral type	53 (41.1%)	28 (71.8%)	1 (9.1%)	17.742	0.001
	Visceral type	68 (52.7%)	11 (28.2%)	9 (81.8%)	12.198	0.002
	Cerebral-visceral type	4 (3.1%)	0 (0.0%)	1 (9.1%)	2.772	0.250
	Bone-muscle type	4 (3.1%)	0 (0.0%)	0 (0.0%)	1.583	0.453
Initial symptom	Neurological dysfunction	53 (41.1%)	28 (71.8%)	2 (18.2%)	15.101	0.001
	Hepatic dysfunction	49 (38.0%)	9 (23.1%)	8 (72.7%)	9.333	0.009
	Renal dysfunction	11 (8.5%)	2 (5.1%)	1 (9.1%)	0.506	0.776
	Bone-musclar dysfunction	4 (3.1%)	0 (0.0%)	0 (0.0%)	1.583	0.453
	Other dysfunction	12 (9.3%)	0 (0.0%)	0 (0.0%)	4.985	0.083

### Details in Timely Diagnosed Group

Among the 11 cases, six were men and five were women, aged 2–62 years, with an average of 14 (5, 38) years. The time from onset to diagnosis was 0.08 (0.04, 0.17) years, and nine cases had the first non-cerebral symptoms (Table 1). Clinical classification: cerebral type 1 case, visceral type 9 cases, cerebral–visceral type 1 case. Initial symptoms: onset of cerebral symptoms in two cases (18.2%, all of which were dysarthria), liver symptoms in eight cases (72.7%, mainly liver dysfunction, jaundice, cirrhosis, splenomegaly, ascites), and kidney symptoms in 1 case (9.1%; Table 2).

### Comparison Among the Three Groups

As indicated in Table 2, the clinical classification of the three groups was mostly cerebral type or visceral type. The clinical misdiagnosis group and the timely diagnosed group accounted for the largest proportion of visceral type (52.7 and 81.8%, respectively), while the undiagnosed group was dominated by cerebral type (71.8%). Only the clinical misdiagnosis group had bone-muscle type (3%) among the three groups. When it comes to initial symptoms, most of the cases in the three groups had similar symptoms, like the cerebral symptoms or liver symptoms which are the common target organs of WD. In particular, the initial symptoms of the undiagnosed group and timely diagnosed group were consistent with their clinical classification, which were cerebral symptoms and liver symptoms, accounting for 71.8 and 72.7%, respectively. The clinical misdiagnosis group had the initial symptoms of cerebral symptoms, accounting for 41.1% and there were no bone–muscle symptoms found in the undiagnosed group and timely diagnosed group.

## Discussion

Wilson's Disease is a systemic disease, also known as autosomal recessive copper metabolism disorder caused by ATP7B gene mutation, leading to extra copper ions deposited in the liver, cerebrum, kidney, cornea, and other tissues because of hepatic copper excretion defect (4). The genotype–phenotype correlations of WD remains elusive. Some studies suggested that compared with H1069Q compound heterozygotes, patients with homozygosity of the H1069Q mutation presented later onset of WD and more neurological dysfunction. Other studies pointed out that frameshift and nonsense mutations in ATP7B leads to lower serum ceruloplasmin levels than those with missense mutations (5). Due to the wide spectrum of WD symptoms, one or more system damage symptoms may occur. So, the initial symptoms are complex and diverse, and lack specificity, which are easily misdiagnosed as various diseases. The onset form of WD can be acute or chronic. As our study shows, patients with acute onset are easily misdiagnosed as acute jaundice or non-icteric hepatitis, hemolytic anemia, upper gastrointestinal hemorrhage, acute nephritis, etc. Chronic onset included chronic hepatitis, cirrhotic ascites, hypersplenism, idiopathic tremor, Parkinson's disease, cerebral atrophy, encephalitis or encephalopathy, various extrapyramidal diseases, etc. Compared with the 51.0% misdiagnosis rate of WD reported by Hu et al. in 2001 (6), 20 years later, 72.1% of the 179 cases we counted were also misdiagnosed for a long time and therefore treatment was delayed. If 21.8% of the patients with unknown diagnosis were added, the proportion of patients who were not diagnosed in time was much higher. Although the number of cases analyzed is small, this data still strongly indicates that WD can mimic many diseases and confuse most doctors. Therefore, to improve the understanding and attention of domestic doctors to WD is particularly crucial.

In this study, 55.8% of patients in the clinical misdiagnosis group had non-cerebral injury symptoms as the first symptom, while only 28.2% of patients in the undiagnosed group had non-cerebral injury symptoms, and the difference between the two groups was significant (χ^2^ = 13.183, *P* < 0.01; Table 1). Tracing the causes, most of the patients with liver, kidney, bone, joint, blood, and other non-cerebral damage as the first symptom often went to the gastroenterology, hematopathology, pediatric, and other departments of local hospitals rather than the neurology department, and the receiving doctors' insufficient understanding of the disease or lack of vigilance resulted in long-term misdiagnosis. However, most of the patients (71.8%) with cerebral symptoms are often undiagnosed for a long time due to lack of neurologist in primary hospitals or poor laboratory conditions.

According to our research, it is worth noting that six patients with mental and psychological abnormality as the first symptom of brain damage were misdiagnosed without exception, with a mean delay in diagnosis being 3.0 years (range: 0.25–9 years). It has been reported that about 30% of patients reported mental symptoms before diagnosis of WD. A study analysis of 58 patients with mental disorder caused by WD showed that the cases with mental symptoms as the first symptom accounted for 29.31% (7). Another study presented 23.5% patients with psychiatric dysfunction, which was misdiagnosed (8). Mura et al. (9) found that about 50%−70% of patients with WD developed psychiatric symptoms, which could not only be manifestations of WD, but concomitant with the type of hepatic, cerebral or hepato-cerebral, or may be the side effects of treatment, which suggests the importance of analyzing psychiatric symptoms in the diagnosis and treatment of WD, and the reasons for misdiagnosis may be as follows. First of all, psychiatric symptoms are more prominent than neurological symptoms in the course of the disease; meanwhile, some extrapyramidal symptoms after antipsychotics are mistaken for adverse reactions and lack of attention. Secondly, psychiatrists pay more attention to psychiatric examinations in clinical work and neglect the collection of neurological history, physical examinations, and necessary auxiliary examinations, such as slit-lamp examination.

As for the relationship between age of onset and misdiagnosis, there was no statistically significant difference between the three groups in the age of onset, which was different from the conclusion of Hu et al. (6), which was related to the sample size. Professor Hu et al. concluded that the clinical misdiagnosis group whose initial symptoms were mainly liver and kidney damage mostly occurred in children, while the undiagnosed group whose initial symptoms were mainly brain damage mostly occurred in young people. Liver disease is the first symptom of up to 60% of patients with WD in all age groups, and can be completely asymptomatic, which may be found by occasional physical examination, or with signs and symptoms of liver disease. Most children present with predominant liver disease, although mild neurological disease may already exist (10). Therefore, the American Association for the Study of Liver Diseases pointed out in the guideline (11) that the possibility of WD should be considered in any patients aged 3–55 with unexplained liver function abnormalities, especially in adolescents.

In the development process of WD, multiple systems and organs tend to be damaged in succession as a result of the differences of abnormal deposition rate, position, and the degree of distribution of copper. For example, the symptoms of nervous system generally occur about 10 years later than liver symptoms (12). As a result, it may easily be long-term misdiagnosed for patients with only a single viscera damage and clinicians appear to consider the diagnosis of WD only when the patient develops secondary organ damage, such as the brain, especially the extrapyramidal system.

Taken together, it is highly possible for patients to be misdiagnosed and mistreated in the early stage of the WD. Therefore, we should be alert to the possibility of WD when patients are acting like the following two aspects. First of all, young patients with unknown cause of abnormal liver function, cirrhosis, splenomegaly, hypersplenism, nephritis, arthritis, hemolytic anemia, mental disorders, encephalopathy (especially extrapyramidal system symptoms) and other diseases, especially when the symptoms of multiple systems damage occur simultaneously or successively, and the therapeutic efficacy is not good, then the WD diagnosis should be considered. Secondly, the diagnostic possibility of WD should be suspicious in patients presenting with family history of jaundice, unexplained childhood death of siblings, or similar symptoms in family. As copper accumulates in Descemet membrane of the cornea, K-F rings occurs, which is the most frequently observed the ocular symptoms of WD. As reported, it happens in 95% of patients with neurological dysfunction and over half of those without neurologic symptoms (13, 14). Compared with the slim lamp, anterior segment optical coherence tomography (OCT), Scheimpflug imaging, and *in vivo* confocal microscopy (IVCM) are new methods to detect K-F rings in WD, especially the IVCM, which can identify subtle structure with high resolution microstructural examination (15). Therefore, we should take this as a key diagnostic feature of WD, which is not only important to the patients themselves, but also one of the screening tests for first-degree relatives of a patient with WD. Thirdly, copper metabolism examination should be performed for diagnosis, which is characterized by obvious decrease in serum total copper content, serum copper oxidase, and serum ceruloplasmin, and significant increase in urinary copper. In imaging examination, abdominal ultrasound and brain magnetic resonance imaging (MRI) are essential to evaluate the liver and brain, especially the basal ganglia region. For most patients with WD, contour irregularity, increased liver echogenicity or periportal thickness, and enlarged spleen were usually found in ultrasound examinations (16). According to statistics, typical brain MRI changes are shown in nearly 100% of untreated patients with WD with neurological dysfunction, including symmetric hyperintensities in T2-weighted images located in the basal ganglia (mainly the putamen and caudate nuclei), thalami, midbrain, and pons. The most interesting sign of brain MRI is regarded as the “face of the giant panda” in the midbrain, which shows in up to 14%−20% of patients with WD with neurological presentations (4, 17, 18). The characteristic pathological changes in the brain of patients with WD are atrophy of the putamen with brown sediment and the transformation of astrocytes, which is called Alzheimer's cells, part of a continuously evolving process, from gradual hypertrophy and proliferation to retrogression (19).

As mentioned above, the diagnosis of WD is dependent on the clinical symptoms score (the Leipzig score), copper metabolism assessment and genetic tests. But, are the genetic tests the golden standard? Antos et al. (20) pointed out that in a 47-year-old female patient who presented atypical manifestation but found two pathogenic variants of ATP7B gene, finally the radioactive copper test was performed to exclude the diagnosis of WD. Radioactive copper test reflects the functional activity of the copper transporter ATP7B, with almost radioactive copper found in the blood of healthy people, while much less can be tested in the patients with WD (21). Therefore, if necessary, radioactive copper may be incorporated as a diagnostic test in doubtful cases.

Wilson's Disease is one of the few genetic diseases that can be treated with good efficacy, which is greatly related to the beginning of treatment. The therapeutic efficacy is surprising if the interval between the initial symptoms and the beginning of treatment is <1 month (22). Therefore, the best course of action is not only required for the clinician to be able to diagnose early but also for parents or patients to detect abnormalities and hospitalize in time. Only with early diagnosis and timely chelation treatment, can most patients obtain long-term clinical remission and maintain a normal life, learning and working skills, as well as similar longevity as a normal person. Otherwise, it may cause irreversible damage to all organs and various serious complications, endangering the patient's life finally (1).

In conclusion, the onset symptom of WD, lacking specificity, is too diversified to be clearly diagnosed. So early diagnosis and correct treatment without delay is of great value to the prognosis. Consequently, as for patients with symptoms described in this article without any certain reasons, especially for youngsters, screening tests including serum copper, ceruloplasmin, corneal K–F ring, urine copper will be indispensable. When necessary, performing complete genetic examination is the most important to detect the WD early among the patient.

## Data Availability Statement

The raw data supporting the conclusions of this article will be made available by the authors, without undue reservation.

## Author Contributions

All authors listed have made a substantial, direct, and intellectual contribution to the work and approved it for publication.

## Conflict of Interest

The authors declare that the research was conducted in the absence of any commercial or financial relationships that could be construed as a potential conflict of interest.

## Publisher's Note

All claims expressed in this article are solely those of the authors and do not necessarily represent those of their affiliated organizations, or those of the publisher, the editors and the reviewers. Any product that may be evaluated in this article, or claim that may be made by its manufacturer, is not guaranteed or endorsed by the publisher.
